# Acute exposure to air pollution is associated with novel changes in blood levels of endothelin-1 and circulating angiogenic cells in young, healthy adults

**DOI:** 10.3934/environsci.2019.4.265

**Published:** 2019-07-01

**Authors:** Jordan Finch, Daniel W. Riggs, Timothy E. O’Toole, C. Arden Pope, Aruni Bhatnagar, Daniel J. Conklin

**Affiliations:** 1 Department of Pharmacology and Toxicology, School of Medicine, University of Louisville, 505 S. Hancock Street, Louisville, KY 40202, USA; 2 Christina Lee Brown Envirome Institute, University of Louisville, 302 E. Muhammad Ali Boulevard, Louisville, KY 40202, USA; 3 Diabetes & Obesity Center, University of Louisville, 580 S. Preston Street, Louisville, KY 40202, USA; 4 Department of Medicine, School of Medicine, University of Louisville, 500 S. Preston Street Louisville, KY 40202, USA; 5 Department of Economics, College of Family, Home, and Social Sciences, Brigham Young University, E 1060 N Street, Provo, UT 84604, USA

**Keywords:** particulate matter, cardiovascular disease, endothelin-1, circulating angiogenic cells, endothelial dysfunction

## Abstract

Acute and chronic exposures to particulate matter (PM_2.5_) air pollution increase the risk for cardiovascular disease (CVD). A hypothesized mechanism linking PM_2.5_ exposure and CVD is the induction of endothelial dysfunction - a key step to increased CVD risk. Although PM_2.5_ exposure is associated with endothelial dysfunction and the vasoconstrictor peptide endothelin-1 (ET-1) is upregulated in endothelial dysfunction, the effects of PM_2.5_ on ET-1 and whether or not ET-1 mediates the downstream effects of PM_2.5_ are unclear. In addition to examining associations between acute changes in ambient PM_2.5_ and circulating levels of ET-1, we also looked at whether changes in ET-1 were associated with changes in markers of vascular health and systemic injury. For example, endothelial function is maintained in part by circulating angiogenic cell (CAC)-mediated repair, and our recent studies show that CACs in humans and mice are decreased by ambient PM_2.5_ exposure. In the current study, we recruited young, healthy adults who were exposed to natural variations in PM_2.5_, and we analyzed associations between PM_2.5_ and circulating levels of ET-1, between ET-1 and CACs, and between ET-1 and other biomarkers of injury using linear regression analyses. Surprisingly, ET-1 levels were negatively associated with PM_2.5_ levels (β = −0.773, *P* = 0.0005), yet, in contrast, positively associated with two CACs: CAC-2 (CD31^+^/CD34^+^/CD45^+^) and CAC-4 (CD31^+^/CD34^+^/CD45^+^/CD133^+^). Interestingly, ET-1 levels were negatively associated with some biomarkers (platelet factor 4, β = −0.148, *P* = 0.0003; triglycerides, β = −0.095, *P* = 0.041) and positively with other biomarkers: albumin (β = 0.035, *P* = 0.006) and IL-lβ (β = 0.082, *P* = 0.012). These findings further reveal the insidious nature of PI_2.5_’s anti-angiogenic effect including a novel relationship between ET-1 and CACs in young adults exposed to acute elevations of air pollution.

## 1 Introduction

Cardiovascular disease (CVD) is the leading cause of morbidity and mortality worldwide, and the risk of cardiovascular events is greatly increased by exposure to air pollution [1]. Exposure to fine particulate matter (particulate matter with aerodynamic diameter of ≤ 2.5 μm [PM_2.5_]) is specifically linked with increased cardiovascular dysfunction [2,3]. Although a number of hypotheses have been proposed to explain the mechanism by which PM_2.5_ exposure leads to negative health outcomes, the development of endothelial dysfunction seems to be a key component of PM_2.5_-induced CVD [2,3]. There is a strong association between endothelial dysfunction and CVD [4], as exemplified by concurrent decreased formation and/or bioactivity of the vasodilator nitric oxide and increased levels and/or activity of the endothelial-derived vasoconstrictor endothelin-1 (ET-1) [4].

ET-1, a potent peptide vasoconstrictor released by endothelial cells, plays a significant role in regulating vascular homeostasis [5]. Plasma ET-1 has been associated with the development of endothelial dysfunction [4], and it has been reported that the production and function of ET-1 and its receptors are upregulated in a number of disease states associated with endothelial dysfunction, including hypertension and atherosclerosis [5–8]. Furthermore, changes in ET-1 have been associated with changes in both inflammatory [9,10] and thrombotic [11] factors, further increasing the potential for the development of endothelial injury and dysfunction. Although increased ET-1 has been implicated in CVD, its relationships with other markers of endothelial injury and repair and with systemic inflammation have been understudied. Likewise, the association between ET-1 and PM_2.5_ remains unclear [12]. Thus, the present study leveraged an established relationship between acute PM_2.5_ exposure, endothelial injury, and circulating angiogenic cells (CACs) by examining alterations in ET-1 following PM_2.5_ exposure [13,14]. In fact, PM_2.5_ exposure suppresses CACs and growth factor levels and simultaneously increases inflammatory markers in young healthy adults that likely primes the endothelium for injury and development of CVD [14–16].

## 2. Materials and methods

### 2.1 Air Pollution monitoring

A cohort of young, healthy nonsmokers naturally exposed to variations in ambient air pollution (range of PM_2.5_ from 6 to 83 μg/m^3^) were recruited from the Provo, Utah area for this longitudinal study that took place between January and early March of 2009 [15]. All participants (n = 16) were between 18 and 30 years of age, were of normal body weight (BMI between 19 and 25), were free of any acute and/or chronic illness or disorder, and were not exposed to mainstream, secondhand, or environmental tobacco smoke at home, work, or school. Upon meeting the enrollment criteria, participants gave written consent for participation in the study before answering a questionnaire to obtain demographic information and baseline characteristics. The participants were divided into two study groups (8 participants each) in order to streamline blood collection and to ensure rapid sample processing and prompt delivery of blood samples overnight to the University of Louisville (Louisville, KY). All research protocols and consent forms were approved by the Institutional Review Board for human subjects at Brigham Young University (IRB study #F08–0289) and carried out in accordance with The Code of Ethics of the World Medical Association.

Daily PM_2.5_ monitoring was conducted by the State of Utah Division of Air Quality according to the U.S. Environmental Protection Agency’s reference method [17] at two sites (North Provo site and Lindon Elementary) located in the Utah Valley. Additional weather data were collected from the National Weather Service as reported from the Salt Lake City International Airport.

The whole Utah Valley region, including Provo, Utah, is subject to winter temperature inversions that cause the development of a stagnant air mass over the valley floor. PM_2.5_ and other emissions become trapped, and residents are exposed to high levels of pollution [14]. PM_2.5_ levels are regionally distributed, as indicated by high agreement (R^2^ = 0.97) between levels measured at North Provo site and Lindon Elementary both before, during, and after exposures [18], and thus, study participants encountered similar levels of ambient PM_2.5_. Blood collections were done before, during, and after an inversion period. The participants each underwent four blood draws: one during a period with high ambient pollution concentrations (PM_2.5_ >40 μg/m^3^), a period with moderate concentrations (PM _2.5_ ≈ 20 to 40 μg/m^3^), and two draws during periods with low concentrations (PM_2.5_ <10 μg/m^3^), which served as baseline levels [15].

### 2.2 Flow cytometry

Eight different CAC populations were identified in these blood samples by flow cytometry as previously published, as first described [19] and as modified [15]. Platelet-leukocyte aggregates (CD41^+^/CD45^+^ events; [PLAs]) were measured using flow cytometry as published [15].

### 2.3 Biochemical analyses

Additional plasma factors including fibrinogen, cholesterol, triglycerides, albumin, and total plasma protein were previously measured using a semi-automated clinical chemistry analyzer (Cobas Mira 5600 Autoanalyzer) [15]. Commercial ELISA kits were used to quantify plasma levels of serum amyloid A (SAA) (Invitrogen; Carlsbad, CA), ET-1, interleukin-6 (IL-6), interleukin-1β (IL-lβ), vascular endothelial growth factor (VEGF), stromal cell-derived factor-1 (SDF-1), and platelet factor 4 (PF-4) (R&D Systems; Minneapolis, MN).

### 2.4 Statistical analyses

The primary statistical approach was regression analysis between ET-1 and PM_2.5_ (24 h average before blood draw x 50 μg/m^3^) controlling for subject-specific fixed effects. Linear regression was used to assess associations between ET-1 and CAC levels or blood and plasma factors. One participant was excluded from all analyses for extreme ET-1 data (greater than 3 SD beyond the mean). Data are expressed as the mean ± SE (or as indicated). All outcome variables were log-transformed for normality. CAC data were normalized to the sample volume [15]. SAS 9.4 software (SAS Institute, Inc.; Cary, NC) was used for all statistical analyses.

## 3 Results

### 3.1 Subject and study characteristics

A summary of the study participants and of the environmental data on days of blood collection is given in Table 1. PM2.5 levels are given as the average of the 24 h prior to each blood draw. Data were separated based on the blood draw for each of the two participant groups (i.e., Gl, G2).

### 3.2 ET-1

**Figure 1.**
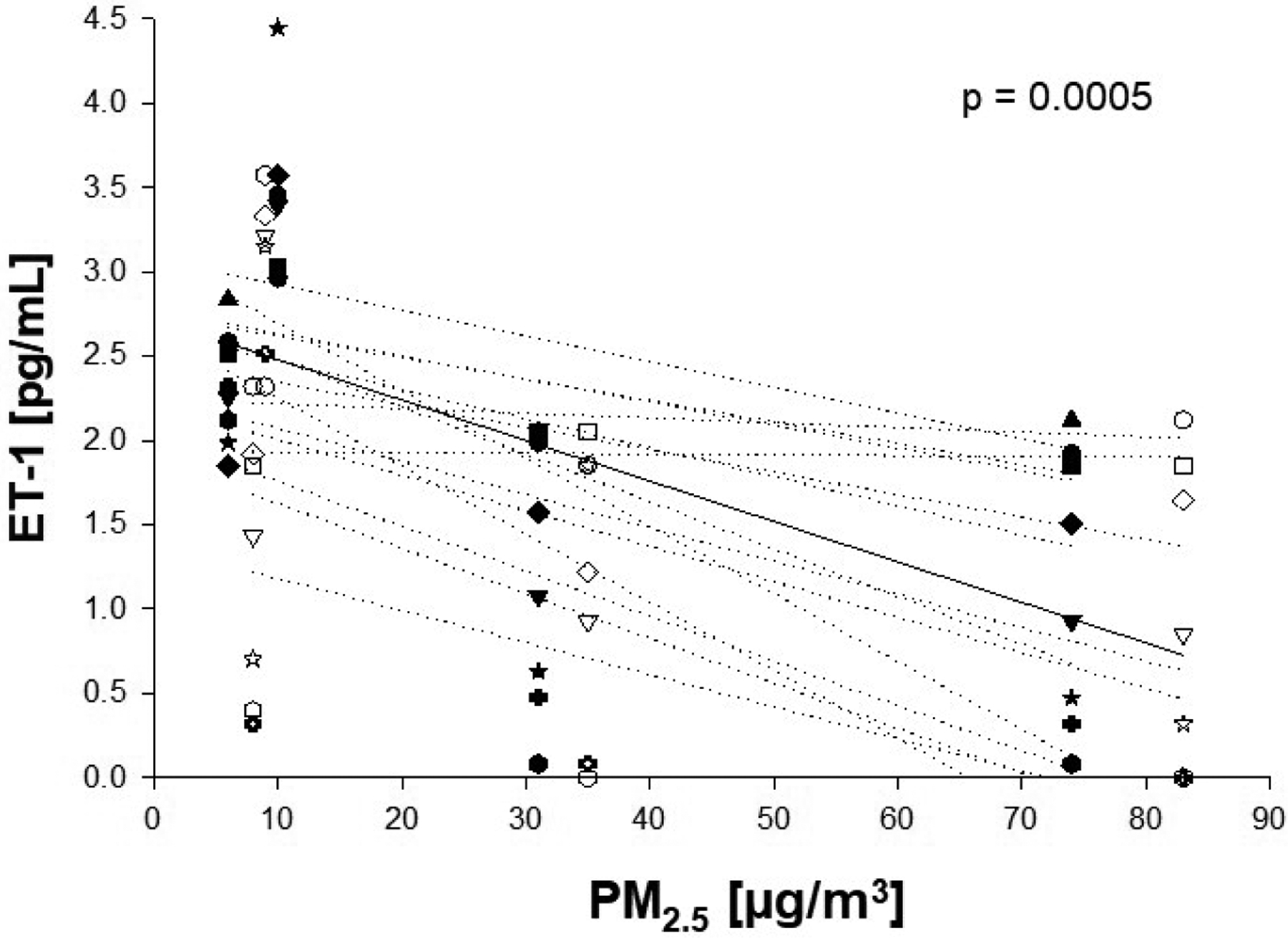
Association between ambient PM_2.5_ and plasma ET-1 levels. Plasma ET-1 levels were regressed on PM_2.5_ (average of 24 h before blood draw x 50 μg/m^3^; see Table 1). A significant association was seen between PM_2.5_ and plasma ET-1 levels. Abbr.: ET-1, endothelin-1; PM, particulate matter. Open symbols = Group 1; Closed symbols = Group 2. Each symbol shape represents a unique individual. Solid line represents predicted mean from fixed effects regression models.

**Figure 2.**
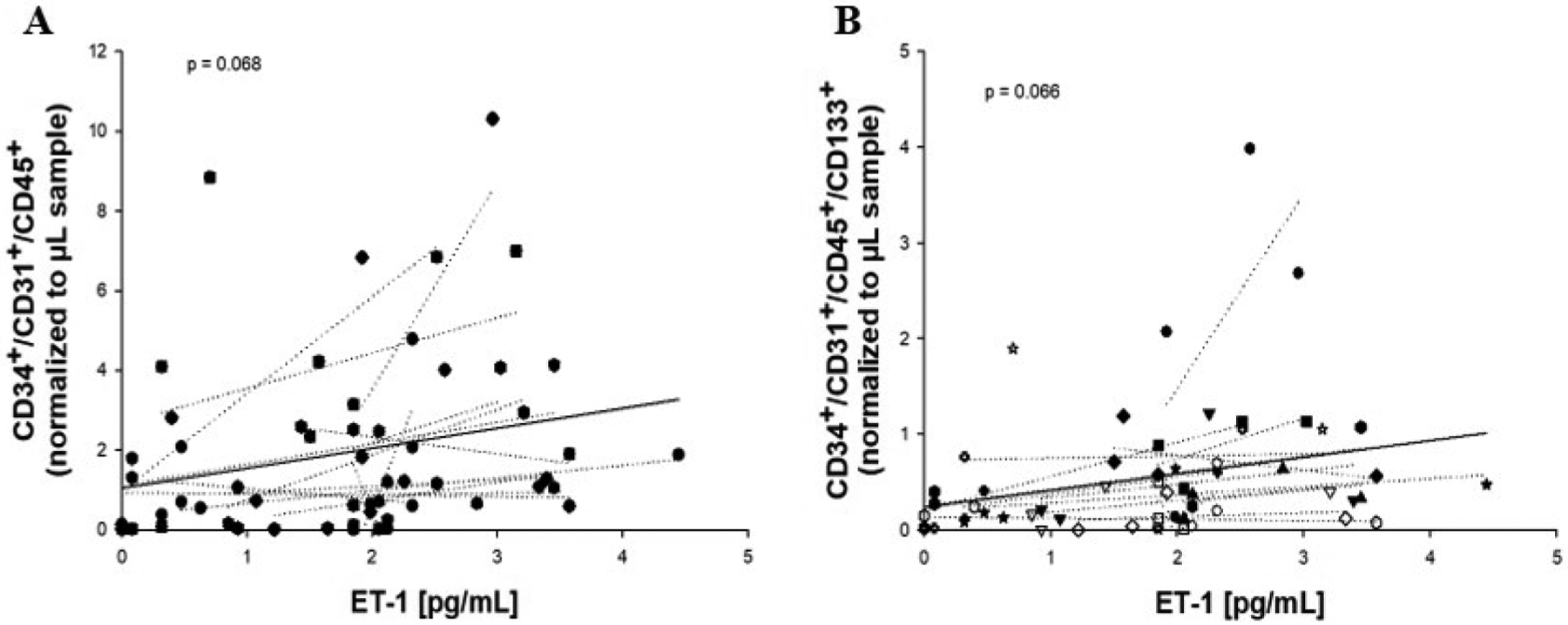
Association between plasma ET-1 levels and CACs. Levels of CACs were regressed on plasma ET-1 levels. Weak, positive associations were seen between ET-1 and CACs 2 (CD34^+^/CD31^+^/CD45^+^) and 4 (CD34^+^/CD31^+^/CD45^+^/CD133^+^). Solid lines represent overall linear regressions. Abbr.: CAC, circulating angiogenic cell, ET-1, endothelin-1.

**Figure 3.**
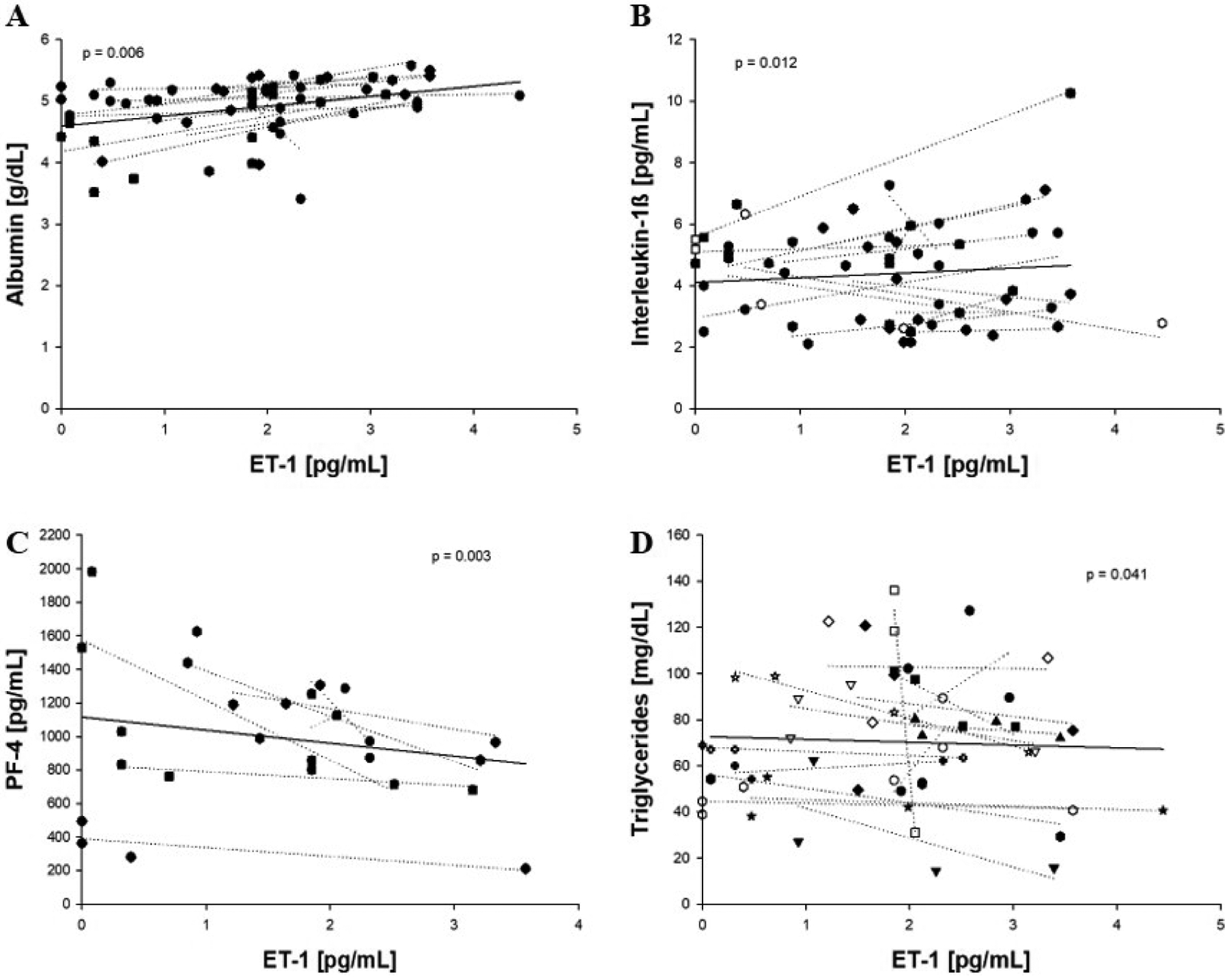
Associations between plasma ET-1 levels and other plasma factors. Levels of plasma factors were regressed on plasma ET-1 levels. Significant positive associations were seen between ET-1 and albumin (A) and interleukin-1β (B), while significant negative associations were seen between ET-1 and PF-4 (C) and triglycerides (D). Abbr.: ET-1: endothelin-1; PF-4: platelet factor 4. Solid lines represent overall linear regressions.

Analysis of the association between ET-1 and PM_2.5_ levels (24 h prior to each blood draw x 50 μg/m^3^) revealed an inverse association between the two factors (β = −0.773; *P* = 0.0005) (Figure 1; Table 2). PM_2.5_ was also analyzed for associations with other previously measured factors [15] except herein with the exclusion of data from a single individual. Significant associations were seen between PM_2.5_ and non-albumin protein, PF-4, SDF-1, and total plasma protein (Table 2; Supplemental Figure 1), consistent with our previous study [15]. Because of the unexpected negative association between PM2.5 and ET-1, and because we previously identified a relationship between PM_2.5_ and CACs [15], further regression analyses were performed to look for associations between ET-1 and CACs. For this, plasma ET-1 levels were used to predict associations with previously measured levels of eight unique CAC populations [15]. Weak, positive associations were present between ET-1 and CAC-2 (CD31^+^/CD34^+^/CD45^+^;β = 0.458, *P* = 0.068) and CAC-4 (CD31^+^/CD34^+^/CD45^+^/CD133^+^; β = 0.402; *P* = 0.066) per 50 μg/m^3^ PM_2.5_ (Figure 2A&B), but no association was observed with the remaining six cell populations (Table 3), suggestive of a specific relationship between ET-1 and these two CAC populations. Regression analyses were also performed to look for associations between ET-1 and previously measured blood and plasma factors [15] with the exclusion of a single outlying individual (Figure 3A–D). Following these analyses, we found that ET-1 was positively associated with albumin and IL-lp yet negatively associated with PF-4 and triglycerides (Table 4). Interestingly, only one plasma factor, PF-4, was associated with levels of both PM_2.5_ (positively) and ET-1 (negatively) (see Tables 2 and 4).

## 4 Discussion

Although several studies report that ET-1 levels increase in response to PM_2.5_ exposure [20–22], others report no change [23,24] or even decreased levels [25]. Our results, like Scharrer et al. [25], found an inverse association between ET-1 and PM_2.5_ exposure levels, with the lowest ET-1 levels corresponding to higher previous 24 h mean PM_2.5_ levels for almost all participants. A likely explanation for these contradictory outcomes is that of acute versus chronic exposure settings. Similar studies [20,22] that report increases in plasma ET-1 have used cohorts that were consistently exposed to high levels of ambient PM_2.5_ throughout the year. As our studies in humans and mice have shown, the acute effects of PM_2.5_ exposure on CACs, for example, are completely reversible in young, healthy adults [15,16]. The nature of reversibility of acute PM_2.5_-induced effects on ET-1 will likely need to be validated in animal exposure studies as well.

Additionally, although we have attempted to ensure the exposure homogeneity of our cohort through our exclusion criteria (e.g., nonsmoking; no smoke exposure at home, work, or school) it is possible that participants were exposed to varying levels of PM_2.5_ during these inversion periods as a result of the amount of time spent outdoors, type of commute, or general physical activity, but we did not capture this variation, which is a limitation of our study. However, we believe that the experimental design of this study (repeated measures) and the large variations in ambient PM_2.5_ levels that occurred during the inversions are strong enough to overcome potential exposure misclassification. Furthermore, the strength of associations observed between ambient PM_2.5_ and circulating factors in this study and as originally reported with this cohort [15] indicates that ambient pollution (PM_2.5_ or a co-varying pollutant) is driving the biological responses. If the relationship between PM_2.5_ and ET-1 were not driven by exposure, we would expect other variations to bring the results towards the null hypothesis rather than showing such a strongly significant association. Findings in our previous animal studies using controlled PM_2.5_ exposures [15,16] also provide the biological plausibility to support our conclusions. Nonetheless, we appreciate that exposure assessment would be enhanced by personal-level monitoring, which could provide additional data that could help refine the relationships between ET-1 and other downstream targets in response to PM_2.5_ exposure.

Although our association study focused on PM_2.5_, it is possible another pollutant(s) (UFP, VOCs) is causal in the observed effects. Calderon et al. [20] found that high levels of ET-1 in elinically-healthy children were associated with 7-day cumulative PM_2.5_ levels, but not with cumulative levels of PM_10_ or ozone. Although these data are contradictory to the negative association between ET-1 and PM_2.5_ seen in our study, this is likely due to the difference in the duration of exposure; the differing outcomes may indicate that episodic exposures to high levels of PM_2.5_ cause decreased levels of ET-1, while persistently-high levels of exposure as seen in Mexico City cause an increase. Nevertheless, the study by Calderon et al. [20] supports our conclusion that PM_2.5_ may influence levels of circulating ET-1. Even so, examining levels of other individual pollutants for associations with ET-1 could help address this discrepancy as well as help to examine the negative health effects seen in healthy individuals in response to high levels of air pollution exposure. These relationships can be explored in future studies.

The biological significance of acutely suppressed ET-1 levels remains to be better clarified. For example, few studies have looked at the relationships between ET-1 and CACs [10,13], and none have looked at these two factors together in a healthy human cohort as in our present study. Although weak, positive associations are observed between CAC-2 (CD31^+^/CD34^+^/CD45^+^) and CAC-4 (CD31^+^/CD34^+^/CD45^+^/CD133^+^) and ET-1 levels, these relationships are likely indirect. Evidence for this idea is supported by Jung et al. [10], who report no change in CAC levels (CD34^+^, CD34^+^/CD133^+^, CD34^+^/KDR^+^, CD34^+^/CD133^+^/KDR^+^) in response to treatment with bosentan, a dual ET_A_/ET_B_ receptor antagonist, which is functionally equivalent to lowering ET-1 levels. Even if the relationship is indirect, suppression of both ET-1 and CAC levels along with a handful of growth factors (VEGF, GRO-alpha, EGF, PDGF-BB) represents an overall “anti-angiogenic” effect of PM_2.5_ exposure that suppresses angiogenesis and endothelial repair [14]. Surprisingly, ET-1 also is pro-angiogenic – both independently and in conjunction with VEGF [26]. Therefore, under these conditions, ET-1 suppression may simply reflect the systemic anti-angiogenic effect of acute PM_2.5_.

As for many of the circulating factors, we cannot distinguish the mechanism regulating the level, which is a function of both formation and clearance, using data from only a single inversion event. Despite this, we did observe several strong associations between ET-1 and other factors that are suggestive of possible mechanisms. For example, we saw a positive association between ET-1 and albumin, an acute phase protein that is transcriptionally decreased with systemic infection [27]. However, the lack of strong association between ET-1 and other inflammatory markers likely indicates that the decreases in albumin (and maybe ET-1) are part of a more subtle change perhaps in endothelial permeability [28] or possibly related to albumin’s antioxidant abilities [29], which may serve as a protective mechanism against PM-induced oxidative stress [3].

ET-1 levels are negatively associated with levels of triglycerides and of PF-4. The few studies reporting on ET-1 and triglycerides show no association between the two markers [30,31], while our study shows an inverse association. This discrepancy may be related to our healthy cohort, as these previous studies have utilized diseased murine models; the lack of confounding health factors in our participants may have allowed for the emergence of this unique association between ET-1 and triglycerides. PF-4 is an indicator of elevated thrombosis, but it also has anti-angiogenic effects [32]. Taken together, these results seem to indicate that PM_2.5_ exposure can induce an anti-angiogenic state even in young, healthy cohorts that could potentially lead to more serious effects in other individuals, such as the development of CVD related to insufficient blood flow, including ischemic heart disease and peripheral vascular disease [33]. We did see an association between ET-1 and IL-1β, an inflammatory cytokine, but this positive relationship indicated that, in this study, these exposures did not trigger a robust systemic inflammatory response.

## 5 Conclusion

In summary, this study examined a potential mediator in the complex mechanism by which air pollution exposure initiates CVD. Our data reveal significant associations between ET-1 and PM_2.5_ and between ET-1 and other markers of vascular change and injury in a young, healthy cohort, thus providing evidence that PM_2.5_ can induce acute changes within the vascular endothelium that reflect disturbances in cardiovascular health. Future studies are required to further investigate both the acute and chronic role of ET-1 in the complex association between air pollution exposure and the development of CVD.

## Supplementary Material

Supplemental Figure 1

## Figures and Tables

**Table 1.** Summary of study design, participant characteristics, and environmental conditions by blood draw and date.

Draw	Draw 1		Draw 2		Draw 3		Draw 4	
Date (mm/dd) (2009)	01/15	01/20	01/22	02/03	02/19	02/24	02/26	03/03
Variable Group	G1	G2	G1	G2	G1	G2	G1	G2
Sex n (%) Female Male	3 (43)	4 (50)	3 (43)	4 (50)	3 (43)	4 (50)	3 (43)	4 (50)
	4 (57)	4 (50)	4 (57)	4 (50)	4 (57)	4 (50)	4 (57)	4 (50)
Age (years ± SE)	22 ± 1	22 ± 0	22 ± 1	22 ± 0	22 ± 1	22 ± 0	22 ± 1	22 ± 0
Environmental Factors								
Temperature (°C)	−3	−4	2	0	1	11	7	15
Relative Humidity (%)	66	61	76	62	51	53	51	26
Barometric Pressure (in Hg)	30.34	30.34	29.93	30.29	30.21	29.84	29.72	29.65
PM_2.5_ (μg/m^3^)	35	74	83	31	8	6	9	10

Notes: Study participants’ characteristics (n = 15 subjects; all Caucasian) and the environmental factors designated by blood draw and date. Values = mean ± SE. Participants were divided into two study groups (G1, G2) for each blood draw. PM_2.5_ levels are given as 24 h average before blood draw.

Abbr.: PM: particulate matter.

**Table 2.** Associations between ambient PM_2.5_ and blood and plasma factors.

	PM_2.5_		
Blood and Plasma Factors	β	p-value	95% Confidence Interval
Albumin (g/dL)	0.015	0.534	−0.032, 0.061
Non-albumin Protein (g/dL)	0.140	<0.001*	0.079, 0.202
Total Plasma Protein (g/dL)	0.058	0.024*	0.008, 0.109
Endothelin-1 (pg/mL)	−0.773	<0.001*	−1.18, −0.365
Fibrinogen (mg/dL)	0.057	0.378	−0.072, 0.187
hsCRP (mg/L)	0.268	0.358	−0.314, 0.849
Interleukin-1β (pg/mL)	0.004	0.941	−0.114, 0.123
Interleukin-6 (pg/mL)	0.119	0.584	−0.319, 0.557
LDL (mg/dL)	0.013	0.367	−0.015, 0.041
PLAs (%CD41^+^/45^+^ cells)	0.232	0.153	−0.090, 0.554
PF-4 (pg/mL)	0.221	0.007*	0.068, 0.373
RBC (x10^6^ cells/μL)	0.008	0.330	−0.008, 0.023
SDF-1 (pg/mL)	−0.067	0.005*	−0.113, −0.022
Serum Amyloid A (μg/mL)	0.068	0.766	−0.393, 0.530
Triglycerides (mg/dL)	−0.055	0.508	−0.219, 0.110
VEGF (pg/mL)	0.087	0.242	−0.063, 0.237
WBC (x10^3^ cells/μL)	0.011	0.713	−0.050, 0.073

Note: Log-transformed outcomes were regressed on PM_2.5_ (average of 24 h before blood draw x 50 μg/m^3^). Values for PF-4 and for VEGF given for participants 1–8 only. Non-albumin protein = Total Plasma Protein – Albumin.

*: significance at the *P* < 0.05 level.

Abbr.: hsCRP: high-sensitivity C-reactive protein; LDL: low density lipoprotein; PF-4: platelet factor 4; PLAs: platelet-leukocyte aggregates; PM: particulate matter; RBC: red blood cells; SDF-1: stromal cell-derived factor-1; VEGF: vascular endothelial growth factor; WBC: white blood cells.

**Table 3.** Associations between plasma ET-1 levels and circulating angiogenic cells.

	Association parameters between CACs & ET-1		
CACs	β	p-value	95% Confidence Interval
CD34^+^/CD31^+^/CD45^−^	0.148	0.511	−0.303, 0.599
CD34^+^/CD31^+^/CD45^+^	0.458	0.068^†^	−0.035, 0.951
CD34^+^/CD31^+^/CD45^−^/CD133^+^	0.010	0.936	−0.232, 0.251
CD34^+^/CD31^+^/CD45^+^/CD133^+^	0.402	0.066^†^	−0.029, 0.833
CD31^+^/CD133^+^	−0.022	0.854	−0.266, 0.221
CD34^+^/CD31^+^	0.281	0.211	−0.165, 0.726
CD34^+^/CD31^+^/CD45^−^/CD133^−^	0.264	0.328	−0.274, 0.802
CD34^+^/CD31^+^/CD45^+^/CD133^−^	0.504	0.259	−0.386, 1.394

Notes: Log-transformed outcomes were regressed on ET-1.

†: significance at 0.05 < *P<* 0.10 level.

Abbr.: CAC: circulating angiogenic cell; ET-1: endothelin-1.

**Table 4.** Associations between plasma ET-1 levels and blood and plasma factors.

	ET-1		
Blood and Plasma Factors	β	p-value	95% Confidence Interval
Albumin (g/dL)	0.035	0.006*	0.011, 0.060
Non albumin Protein (g/dL)	−0.016	0.450	−0.059, 0.027
Fibrinogen (mg/dL)	−0.022	0.358	−0.094, 0.050
hsCRP (mg/L)	0.260	0.118	−0.068, 0.588
Interleukin-1β (pg/mL)	0.082	0.012*	0.019, 0.146
Interleukin-6 (pg/mL)	0.135	0.243	−0.096, 0.366
LDL (mg/dL)	0.006	0.430	−0.010, 0.023
PLAs (% total of CD41^+^/45^+^ cells)	0.170	0.068	−0.013, 0.352
PF-4 (pg/mL)^&^	−0.148	0.003*	−0.238, −0.059
RBCs (x10^6^ cells/μL)	−0.002	0.653	−0.011, 0.007
SDF-1 (pg/mL)	0.021	0.131	−0.007, 0.049
Serum Amyloid A (μg/mL)	0.075	0.573	−0.190, 0.340
Total Plasma Protein (g/dL)	0.018	0.243	−0.013, 0.048
Triglycerides (mg/dL)	−0.095	0.041*	−0.185, −0.004
VEGF (pg/mL) ^&^	−0.032	0.482	−0.127, 0.062
WBCs (x10^3^ cells/μL)	−0.009	0.613	−0.044, 0.026

Notes: Log-transformed outcomes were regressed on ET-1. Values for PF-4 and for VEGF given for participants 1–8 only.

*: significance at *P* <0.05 level,

†: significance at 0.05 *<P* < 0.10 level.

Abbr: ET-1, endothelin-1; hsCRP: high-sensitivity C-reactive protein; LDL: low density lipoprotein; PF-4: platelet factor 4; PLAs: platelet-leukocyte aggregates; RBC: red blood cell; SDF-1: stromal cell-derived factor-1; VEGF: vascular endothelial growth factor; WBC: white blood cell.

## Highlights:

- Increased particulate matter was negatively associated with levels of endothelin-1.
- Endothelin-1 levels were associated with levels of specific circulating stem cells.

## Abbreviations:

CACs: circulating angiogenic cells
CVD: cardiovascular disease
ET-1: endothelin-1
hsCRP: high-sensitivity C-reactive protein
IL-lβ: interleukin-lβ
IL-6: interleukin-6
LDL: low density lipoprotein
PF-4: platelet factor 4
PLAs: platelet-leukocyte aggregates
PM: particulate matter
RBC: red blood cells
SAA: serum amyloid A
SDF-1: stromal cell-derived factor-1
VEGF: vascular endothelial growth factor
WBC: white blood cells

## References

[1] World Health Organization, Health topics: air pollution. World Health Organization, 2014 Available from: www.who.int.

[2] Pope CA, Dockery DW (2006) Health Effects of Fine Particulate Air Pollution: Lines that Connect. J Air Waste Manage Assoc 56: 709–742. 10.1080/10473289.2006.10464485 16805397

[3] Brook RD, Rajagopalan S, Pope CA, (2010) Particulate matter air pollution and cardiovascular disease: An update to the scientific statement from the American Heart Association. Circulation 121: 2331–2378. 20458016 10.1161/CIR.0b013e3181dbece1

[4] Lerman A, Zeiher AM (2005) Endothelial Function: Cardiac Events. Circulation 111: 363–368. 15668353 10.1161/01.CIR.0000153339.27064.14

[5] Miyauchi T, Tomobe Y, Shiba R, (1990) Involvement of endothelin in the regulation of human vascular tonus. Potent vasoconstrictor effect and existence in endothelial cells. Circulation 81: 1874–1880. 2188755 10.1161/01.cir.81.6.1874

[6] Vincent R, Kumarathasan P, Goegan P, (2001) Inhalation toxicology of urban ambient particulate matter: acute cardiovascular effects in rats. Res Rep Health Eff Inst 104: 5–54. 11833973

[7] Bohm F, Johansson BL, Hedin U, (2002) Enhanced vasoconstrictor effect of big endothelin-1 in patients with atherosclerosis: relation to conversion to endothelin-1. Atherosclerosis 160: 215–222. 11755940 10.1016/s0021-9150(01)00564-0

[8] Auchincloss AH, Diez Roux AV, Dvonch JT, (2008) Associations between recent exposure to ambient fine particulate matter and blood pressure in the Multi-ethnic Study of Atherosclerosis (MESA). Environ Health Perspect 116: 486–491. 18414631 10.1289/ehp.10899 PMC2291007

[9] Riediker M, Cascio WE, Griggs TR, (2004) Particulate matter exposure in cars is associated with cardiovascular effects in healthy young men. Am J Respir Crit Care Med 169: 934–940. 14962820 10.1164/rccm.200310-1463OC

[10] Jung C, Rafnsson A, Brismar K, (2013) Endothelial progenitor cells in relation to endothelin-1 and endothelin receptor blockade: a randomized, controlled trial. Int J Cardiol 168: 1017–1022. 23168014 10.1016/j.ijcard.2012.10.032

[11] Joseph R, Scicli AG, Han E, (1991) Endothelin-1 and human platelet activity. Thromb Res 61: 529–536. 2028454 10.1016/0049-3848(91)90160-x

[12] Wu S, Yang D, Pan L, (2016) Chemical constituents and sources of ambient particulate air pollution and biomarkers of endothelial function in a panel of healthy adults in Beijing, China. Sci Total Environ 560–561: 141–149. 10.1016/j.scitotenv.2016.03.228 27101449

[13] Paczkowska E, Gołąb-Janowska M, Bajer-Czajkowska A, (2013) Increased circulating endothelial progenitor cells in patients with haemorrhagic and ischaemic stroke: The role of Endothelin-1. J Neurol Sci 325: 90–99. 23290569 10.1016/j.jns.2012.12.005

[14] Pope CA, Bhatnagar A, McCracken JP, (2016) Exposure to Fine Particulate Air Pollution Is Associated With Endothelial Injury and Systemic Inflammation. CircRes 119: 1204–1214. 10.1161/CIRCRESAHA.116.309279 PMC5215745 27780829

[15] O’Toole TE, Hellmann J, Wheat L, (2010) Episodic exposure to fine particulate air pollution decreases circulating levels of endothelial progenitor cells. CircRes 107: 200–203. 10.1161/CIRCRESAHA.110.222679 PMC2943671 20595651

[16] Haberzettl P, Lee J, Duggineni D, (2012) Exposure to Ambient Air Fine Particulate Matter Prevents VEGF-Induced Mobilization of Endothelial Progenitor Cells from the Bone Marrow. Environ Health Per spect 120: 848–856. 10.1289/ehp.1104206 PMC3385427 22418586

[17] United States Environmental Protection Agency (1997) Revised requirements for designation of reference and equivalent methods for PM2.5 and ambient air quality surveillance for particulate matter, Final Rule. In: Monitoring and Quality Assurance Group E, Monitoring, and Analysis Division, editor. Research Triangle Park, NC: Office of Research and Development.

[18] Utah Department of Environmental Quality: Utah Divison of Air Quality, Particulate Matter PM 2.5 Data Archive. Utah Department of Environmental Quality: Utah Divison of Air Quality, 2019 Available from: http://www.airmonitoring.utah.gov/dataarchive/archpm25.htm.

[19] Duda DG, Cohen KS, Scadden DT, (2007) A protocol for phenotypic detection and enumeration of circulating endothelial cells and circulating progenitor cells in human blood. Nat Protoc 2: 805–810. 17446880 10.1038/nprot.2007.111 PMC2686125

[20] Calderon-Garciduenas L, Vincent R, Mora-Tiscareno A, (2007) Elevated plasma endothelin-1 and pulmonary arterial pressure in children exposed to air pollution. Environ Health Per spec t 115: 1248–1253. 10.1289/ehp.9641 PMC1940106 17687455

[21] Lund AK, Lucero J, Lucas S, (2009) Vehicular emissions induce vascular MMP-9 expression and activity associated with endothelin-1-mediated pathways. Arterioscler Thromb Vase Biol 29: 511–517. 10.1161/ATVBAHA.108.176107 PMC4103743 19150882

[22] Chen R, Li H, Cai J, (2018) Fine Particulate Air Pollution and the Expression of microRNAs and Circulating Cytokines Relevant to Inflammation, Coagulation, and Vasoconstriction. Environ Health Perspect 126: 017007. 29342453 10.1289/EHP1447 PMC6014692

[23] Mills NL, Tornqvist H, Robinson SD, (2005) Diesel exhaust inhalation causes vascular dysfunction and impaired endogenous fibrinolysis. Circulation 112: 3930–3936. 16365212 10.1161/CIRCULATIONAHA.105.588962

[24] Langrish JP, Lundback M, Mills NL, (2009) Contribution of endothelin 1 to the vascular effects of diesel exhaust inhalation in humans. Hypertension 54: 910–915. 19687345 10.1161/HYPERTENSIONAHA.109.135947

[25] Scharrer E, Hessel H, Kronseder A, (2007) Heart rate variability, hemostatic and acute inflammatory blood parameters in healthy adults after short-term exposure to welding fume. Int Arch Occup Environ Health 80: 265–272. 16791613 10.1007/s00420-006-0127-2

[26] Salani D, Taraboletti G, Rosanὸ L, (2000) Endothelin-1 Induces an Angiogenic Phenotype in Cultured Endothelial Cells and Stimulates Neovascularization In Vivo. Am JPathol 157: 1703–1711. 11073829 10.1016/S0002-9440(10)64807-9 PMC1885730

[27] Gruys E, Toussaint MJ, Niewold TA, (2005) Acute phase reaction and acute phase proteins. J Zhejiang Univ Sci B 6: 1045–1056. 16252337 10.1631/jzus.2005.B1045 PMC1390650

[28] Lynch JJ, Ferro TJ, Blumenstock FA, (1990) Increased endothelial albumin permeability mediated by protein kinase C activation. J Clin Invest 85: 1991–1998. 2347922 10.1172/JCI114663 PMC296668

[29] Roche M, Rondeau P, Singh NR, (2008) The antioxidant properties of serum albumin. FEBS Letters 582: 1783–1787. 18474236 10.1016/j.febslet.2008.04.057

[30] Horio T, Kohno M, Murakawa K-i, (1991) Increased plasma immunoreactive endothelin-1 concentration in hypercholesterolemic rats. Atherosclerosis 89: 239–246. 1793451 10.1016/0021-9150(91)90065-b

[31] Martinez-Miguel P, Raoch V, Zaragoza C, (2009) Endothelin-converting enzyme-1 increases in atherosclerotic mice: potential role of oxidized low density lipoproteins. J Lipid Res 50: 364–375. 18997155 10.1194/jlr.M800215-JLR200

[32] Maurer AM, Zhou B, Han ZC (2006) Roles of platelet factor 4 in hematopoiesis and angiogenesis. Growth Factors 24: 242–252. 17381065 10.1080/08977190600988225

[33] Ng Y-S, D’Amore PA (2001) Therapeutic angiogenesis for cardiovascular disease. Curr Control Trials Cardiovasc Med 2: 278–285. 11806814 10.1186/cvm-2-6-278 PMC64829

