# Supplementary material for: Acute exposure to air pollution is associated with novel changes in blood levels of endothelin-1 and circulating angiogenic cells in young, healthy adults
Source: AIMS Environ Sci. Author manuscript; Available in PMC 2020 Apr 17. (PMC7164546; doi:10.3934/environsci.2019.4.265)
Supplement: Supplemental Figure 1 [file NIHMS1559708-supplement-Supplemental_Figure_1.docx]

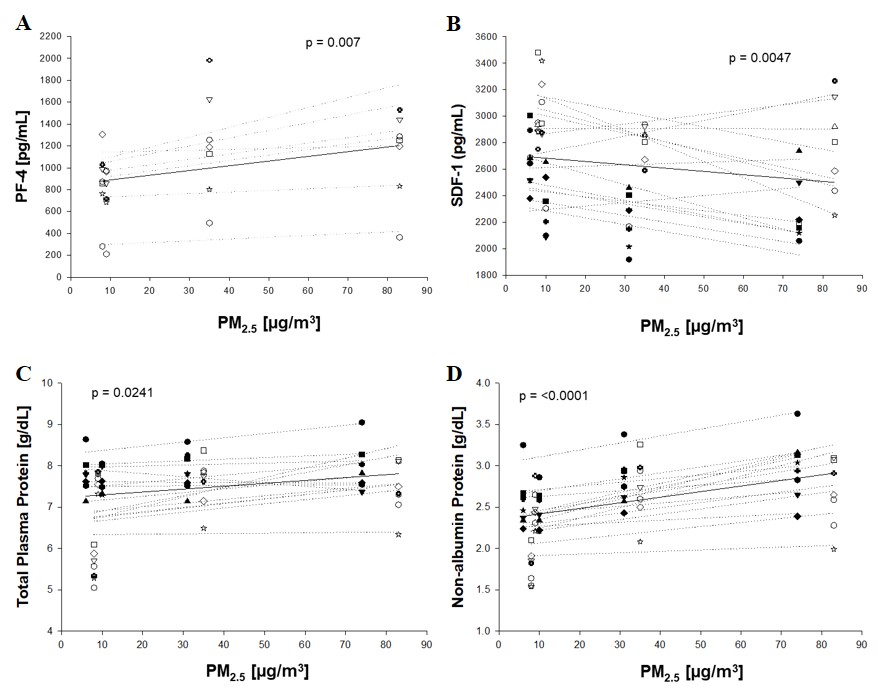


**Supplemental Figure 1:** ***Reanalysis of associations between ambient PM_2.5_ and plasma factors****.* Outcomes were regressed on PM_2.5_ (average of 24h before blood draw; see Table 1) as previously reported with the exclusion of a single outlying individual. Significant associations were seen between PM_2.5_ and several plasma factors, specifically PF-4 (A), SDF-1 (B), total plasma protein (C), and non-albumin protein (D). Abbr.: **PF-4**, platelet factor 4; **PM,** particulate matter; **SDF-1**, stromal cell-derived factor-1. Solid lines represent predicted mean from fixed effects regression models.
